# ECG Vertex-Time Activation Representation for IoT-XR Heart Monitoring

**DOI:** 10.3390/s26196230

**Published:** 2026-09-30

**Authors:** Aurora Silvi, Edo Fejzic, Eleonora Di Salvo, Stefania Colonnese

**Affiliations:** Department of Information Engineering, Electronics and Telecommunications, Sapienza University of Rome, 00184 Rome, Italy

**Keywords:** ECG monitoring, Internet of Things, Extended Reality, graph signal processing, low-latency IoT systems

## Abstract

Electrocardiogram (ECG) monitoring via wearable devices generates rich physiological data, yet conventional time-domain representations offer limited support for spatial interpretation and immersive visualization. We introduce a novel vertex-time representation of the ECG signal, introducing the Ideal Vertex Time (IVT) and Eigen Vertex Time (EVT) vectors which map temporal activation intervals and eigenbeat amplitudes onto a three-dimensional heart point cloud. A key novelty is that this approach serves a dual purpose: it enables graph-spectral ECG feature extraction for classification tasks, and it drives immersive rendering within an Internet of Things pipeline, based on MQTT, cloud processing, and Extended Reality visualization. This work is presented as a proof of concept. The mapping from ECG landmarks to anatomical regions is model-driven and has not been independently validated against patient-specific electrophysiological measurements. Herein, the framework is evaluated for classification purposes on an affective-state dataset and integrated into an Internet-of-Things-enabled e-health monitoring pipeline, whose prototype replays previously recorded ECG signals rather than acquiring them from a wearable device. Overall, this work extends conventional time-domain electrocardiographic analysis toward a spatially structured and immersive monitoring framework. No comparison with conventional ECG descriptors was performed; thus, no advantage over such features is claimed. Its clinical utility remains to be assessed within a patient-specific monitoring pipeline, computing the vertex-time features alongside conventional ECG descriptors and weighting them jointly to identify the best representation for each patient.

## 1. Introduction

Heart activity monitoring plays a crucial role in applications as preventive healthcare or emotion classification. Wearable devices for acquiring electrocardiogram (ECG) signals enable monitoring outside clinical environments, fostering patient-centric solutions [1,2,3].

Despite the availability of long-term ECG streams, extracting meaningful and interpretable information in real time remains challenging. Traditional ECG analysis relies mainly on time- and frequency-domain descriptors [4], while emerging applications such as digital twins and immersive monitoring require representations that are both computationally efficient and intuitively understandable [5,6,7].

In parallel, Extended Reality (XR) technologies are increasingly being adopted in healthcare for visualization, training, and monitoring. XR allows complex physiological processes to be represented dynamically and spatially, improving interpretability and user engagement [8,9,10].

From a signal-processing perspective, recent studies have highlighted the importance of non-Euclidean representations, such as graphs and point clouds, for modeling cardiac activity. These representations naturally capture multiscale dependencies [11] and structural relationships that are difficult to express using conventional features [12,13,14,15]. Additionally, recent advances in intelligent rehabilitation and human–computer interaction have also highlighted the potential of lightweight learning architectures and three-dimensional graph-based representations for healthcare-oriented applications [16,17].

Existing ECG monitoring solutions generally process physiological signals in the time or frequency domain and use XR primarily as a visualization layer. Conversely, graph-based cardiac representations often focus on anatomical or electrophysiological modeling without integrating the resulting features into an end-to-end IoT-XR communication pipeline.

Herein, we address this gap in the literature, and we introduce a model-driven vertex-time framework that maps ECG-derived information onto a three-dimensional heart point cloud, describing the ECG from a spatio-temporal point of view. This approach paves the way for vertex-time feature extraction and immersive visualization. Specifically, we define the Ideal Vertex Time (IVT) and Eigen Vertex Time (EVT) graph-based ECG representations, which map temporal ECG components onto a predefined three-dimensional heart geometry. This mapping enables vertex-time graph-spectral analysis. The representations are evaluated in proof-of-concept classification experiments and integrated into an IoT-XR prototype to demonstrate the feasibility of immersive cardiac visualization.

The main contributions of this paper are as follows:

- The definition of the ECG-derived vertex-time signal through two different representations: IVT, which encodes trigger, depolarization, and repolarization intervals, and EVT, which maps eigenbeat amplitudes onto predefined anatomical regions of a three-dimensional heart point cloud.
- The formulation of a graph-spectral processing framework in which the cardiac geometry is modeled as a weighted graph and the Graph Fourier Transform is applied to the IVT and EVT signals to extract low- and high-frequency coefficients.
- A proof-of-concept experimental evaluation of IVT, EVT, and combined IVT + EVT features for valence and arousal classification, including the analysis of different spectral configurations and heart-mesh resolutions.
- The integration of the proposed vertex-time representations into an end-to-end IoT-XR prototype based on MQTT communication, Node-RED middleware, cloud processing, and web-based immersive visualization.

**Figure 1:**
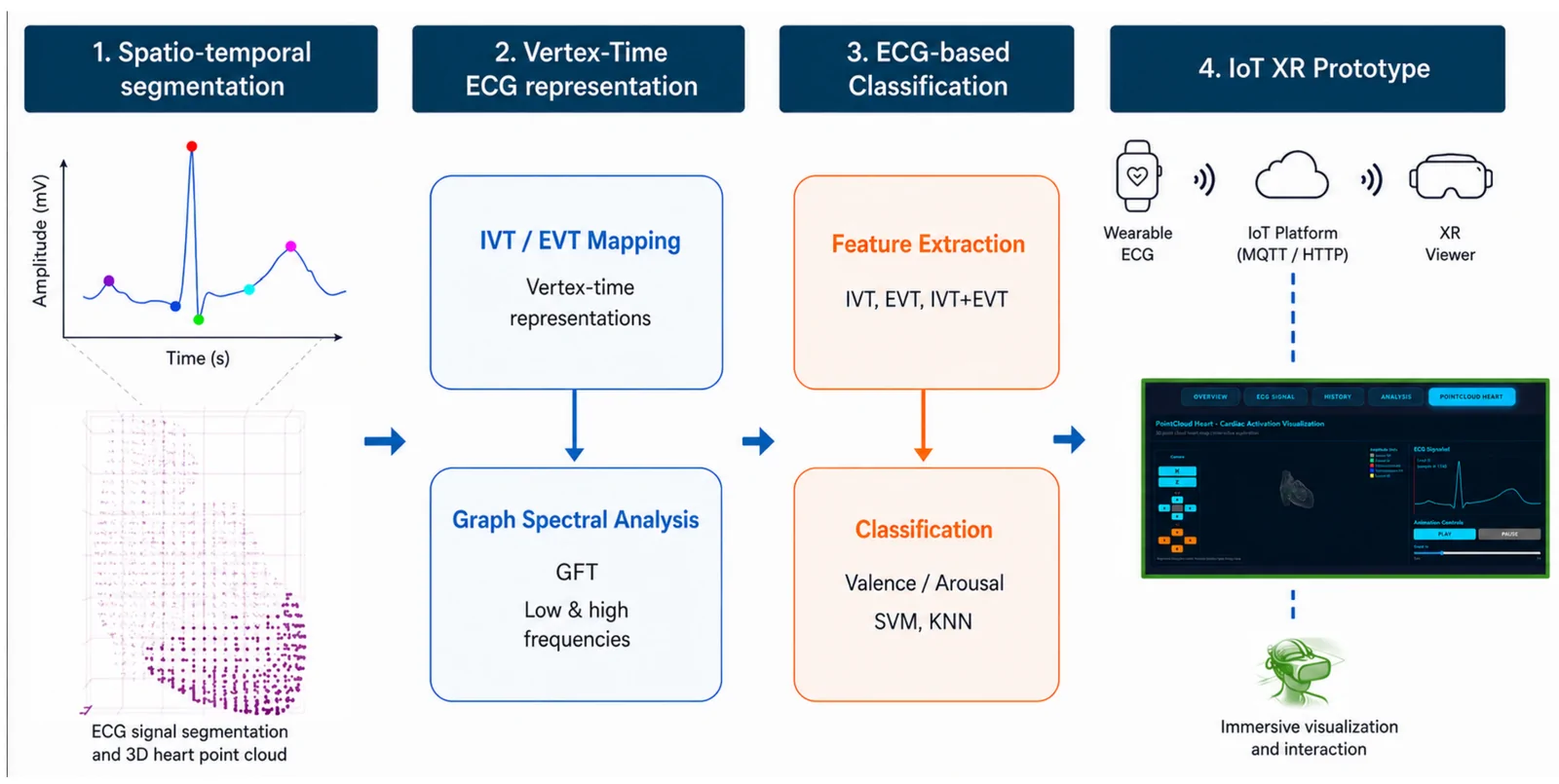
Overview of the proposed framework, from ECG spatio-temporal segmentation and IVT/EVT representation to graph-spectral analysis, affective-state classification, and IoT-XR visualization.

The methodological, experimental, and implementation limitations of the current proof-of-concept framework are explicitly discussed, together with the requirements for future participant-independent, patient-specific, and clinical validation. The remainder of this paper is organized as follows. Section 2 reviews wearable ECG monitoring, non-Euclidean cardiac representations, XR applications in cardiology, and IoT-based ECG streaming architectures. Section 3 introduces the vertex-time ECG representation, including ECG preprocessing, anatomical mapping, and graph-spectral feature extraction. Section 4 presents the classification experiments, the experimental protocols, and the analysis of spectral and geometric configurations. Section 5 describes the end-to-end IoT-XR prototype, from ECG streaming to immersive visualization. Section 6 discusses the methodological, experimental, and implementation limitations, while Section 7 concludes the paper. An overview of the complete proposed framework, from ECG processing and vertex-time representation to classification and IoT-XR visualization, is shown in Figure 1.

## 2. Related Work

Wearable ECG monitoring systems enable continuous, out-of-hospital monitoring of cardiac activity and have demonstrated clinical potential for long-term ECG acquisition, heart rate estimation, and arrhythmia screening. Recent studies confirm their feasibility and diagnostic value using both smartwatches and multi-lead recordings, including the identification of ST-segment changes compared to standard 12-lead ECG [1,18].

Beyond conventional temporal and spectral analysis, non-Euclidean ECG representations have been proposed to capture multiscale dependencies and the spatial organization of cardiac activity. In particular, graph-based models exploiting wavelet features have shown robustness in affective computing and physiological state classification, while point-cloud and mesh-based cardiac representations enable the modeling of activation patterns over realistic anatomical geometries [12,13,14]. These approaches support the extraction of both geometric and spectral features directly on 3D heart models.

In parallel, XR has gained increasing relevance in cardiology. A systematic review covering publications up to mid-2024 identified 164 XR-related studies in cardiology, with applications ranging from medical education to intra-procedural support [8]. Because this field is rapidly evolving, we complement that review with more recent studies published in 2025 and 2026 addressing real-time XR interaction, spatial cardiac visualization, and multimodal clinical-data integration [9,19,20].

The integration of ECG processing within XR environments has become an active research area. Augmented Reality (AR)-based solutions have been proposed for arrhythmia classification and interactive 3D cardiac visualization, both in prototypes [21] and in advanced frameworks combining spatial mapping and real-time XR interaction [19]. Additionally, immersive platforms have been designed to support multimodal clinical data visualization for real-time interpretation [20]. Systems for hemodynamic inspection and vascular geometry analysis within XR setups have been developed [22]. For ECG data collection and low-latency processing, Internet of Things (IoT) ECG pipelines commonly adopt lightweight communication protocols and modular middleware. In particular, MQTT-based acquisition effectively manages heterogeneous sensor streams and provides real-time connectivity to visualization layers [5,10]. Previous studies [23] focused on IoT-related challenges and end-to-end latency characterization for ECG-to-XR streaming, primarily using immersive environments as visualization interfaces for ECG traces, classification outputs, anatomical models, or multimodal clinical information. In contrast, the present work introduces ECG-derived vertex-time signals that can be adopted both for physiological feature extraction, e.g., for classification purposes, and for immersive visualization.

Table 1 summarizes representative approaches and highlights their main strengths and limitations with respect to the proposed framework. The comparison shows that existing approaches provide strong solutions for different aspects of the problem, including ECG classification, anatomical modeling, and immersive clinical visualization. We recognize that the proposed framework differs from the literature in introducing the ECG vertex-time representation, jointly enabling graph processing and IoT-XR immersive visualization.

## 3. Vertex-Time ECG Representation

### 3.1. Method Overview

**Figure 2:**
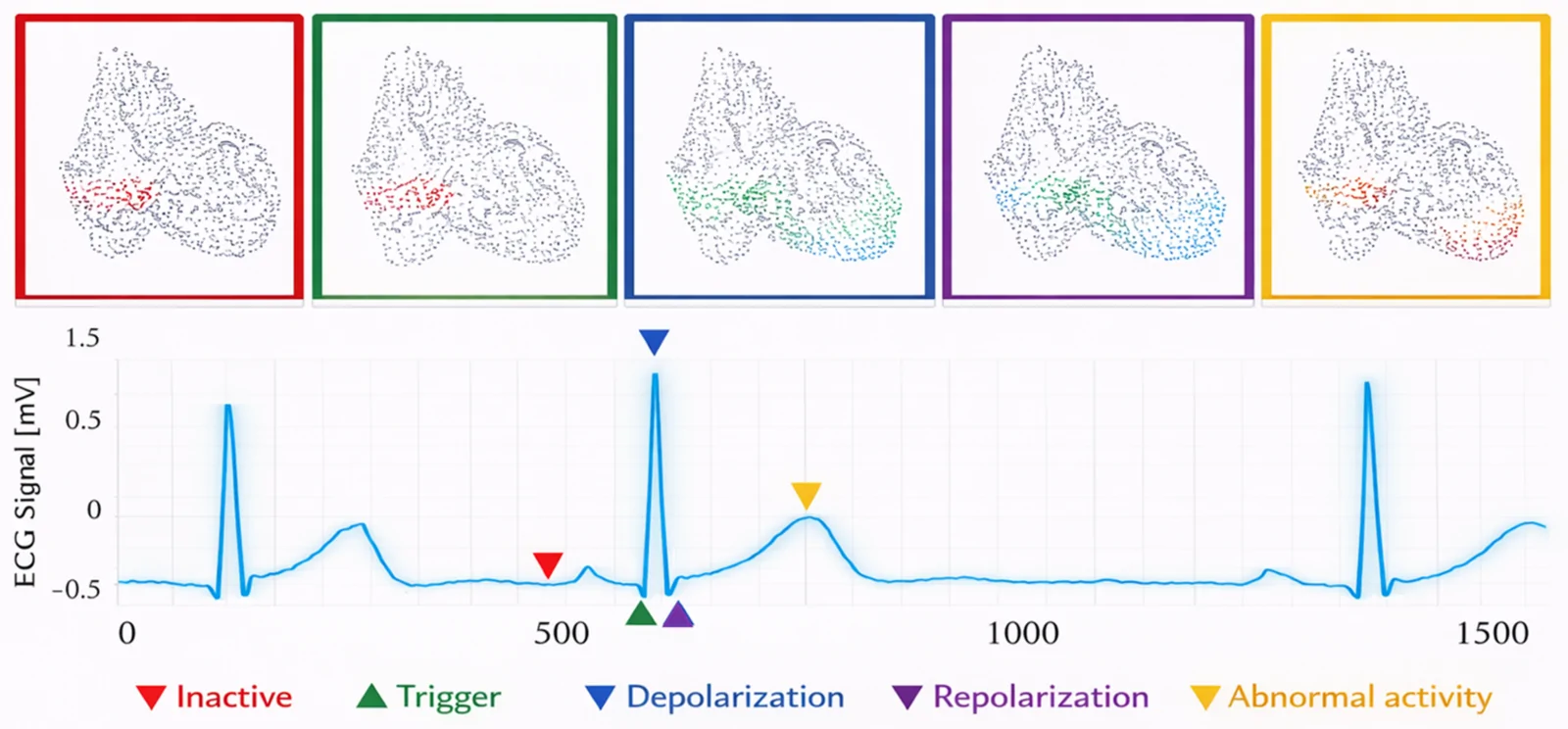
Model-driven mapping of ECG dynamics onto the three-dimensional heart point cloud. The eigenbeat landmarks identify the trigger, depolarization, and repolarization intervals, which are associated with predefined anatomical regions to generate the vertex-time cardiac representation.

The proposed processing chain maps the ECG signal into a spatio-temporal representation; specifically, we introduce graph-based vertex-time signals defined over a three-dimensional heart point cloud. In order to build the spatio-temporal representation, the ECG beats are preliminarily segmented and aligned to estimate the temporal landmarks (P-QRS-T intervals) of the eigenbeat [4]. Temporal ECG intervals are then associated with predefined anatomical regions of the cardiac geometry, producing the IVT and EVT representations. The resulting graph signals are processed through the Graph Fourier Transform, and selected spectral features are extracted for classification. The vertex-time representation is also integrated into a IoT-XR visualization pipeline. Figure 2 summarizes the ECG-to-geometry mapping, where the vertex-time representation is constructed through four stages: (i) temporal segmentation of the ECG signal, (ii) functional spatial segmentation of the heart point cloud, and (iii) algebraic construction of the vertex-time ECG signals, finally leading to (iv) XR visualization.

### 3.2. ECG Signal Temporal Segmentation

For temporal segmentation, given an ECG recording of duration *T*, individual heartbeats are detected, segmented, and aligned at the R peak. The aligned beats are arranged into a matrix whose rows correspond to individual beats and whose columns correspond to time samples. A robust representative waveform, referred to as the eigenbeat ${\hat{s}}_{\mathrm{beat}}$, is then extracted from this matrix using $L_{1}$-norm principal component analysis [4].

The ECG landmarks are identified through the following processing pipeline:

1. Automatic lead selection to improve the robustness of R-peak detection, for multi-lead ECG recordings;
2. QRS landmark identification, including the Q, R, and S points, using a Pan–Tompkins-based approach [24], followed by temporal and physiological consistency checks;
3. Beat segmentation according to physiological timing constraints, including the PQ interval, QRS complex, and ST interval.

**Figure 3:**
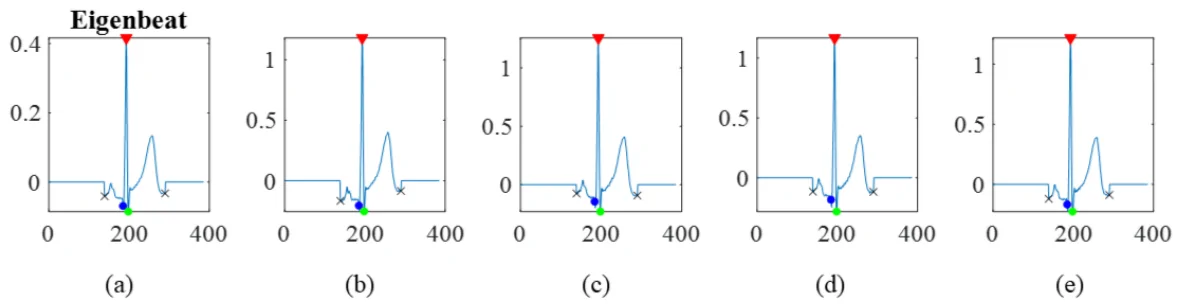
$L_{1}$-PCA eigenbeat estimation and ECG landmark detection. Panel (**a**) presents the representative eigenbeat obtained from the aligned ECG beats, whereas panels (**b**–**e**) report four examples of the aligned beats used for its estimation. Black crosses indicate the P-wave onset and T-wave offset, the blue circle identifies the Q point, the red triangle identifies the R peak, and the green circle identifies the S point. The variability among panels (**b**–**e**) reflects beat-to-beat differences in morphology and amplitude, whereas panel (**a**) captures their robust common component.

The resulting representative eigenbeat and the detected ECG landmarks are illustrated in Figure 3.

To ensure temporal consistency, the detected beats are aligned with respect to the R peak through zero-padding, using the R peak as a common temporal reference.

The resulting eigenbeat captures the common ECG morphology while reducing beat-to-beat variability. Its automatically detected landmarks, including P-wave onset, QRS onset/Q point, R peak, S point, and T-wave offset, provide a compact temporal parameterization of the cardiac cycle. Additional landmarks, such as the P peak, T-wave onset, and T-wave peak, are refined in Figure the subsequent vertex-time analysis.

### 3.3. Heart Point-Cloud Spatial Segmentation

For spatial segmentation, a segmented heart point cloud is considered, where the number of the anatomical regions of the heart (ARH) is denoted as $N_{\mathrm{ARH}}$. To represent the segmentation of the heart point cloud, we introduce the activation space matrix ($B_{\mathrm{space}}$), which is a binary matrix with $N_{\mathrm{PC}}$ rows and $N_{\mathrm{ARH}}$ columns defined in (1), where the *i*-th row refers to the *i*-th point $p_{i}$, and the (i,j)-th coefficient $b_{j}^{\left(\mathrm{space}\right)}\left(p_{i}\right)$ of $B_{\mathrm{space}}$ are defined in (2) and in (3), respectively.

(1) $$B_{\mathrm{space}}=\left[b_{1}^{\left(\mathrm{space}\right)},\ldots ,b_{N_{\mathrm{ARH}}}^{\left(\mathrm{space}\right)}\right]$$

(2) $$b_{j}^{\left(\mathrm{space}\right)}={\left[b_{j}^{\left(\mathrm{space}\right)}\left(p_{1}\right),\ldots ,b_{j}^{\left(\mathrm{space}\right)}\left(p_{N_{\mathrm{PC}}}\right)\right]}^{T};$$

(3) $$b_{j}^{\left(\mathrm{space}\right)}\left(p_{i}\right)=\left\{\begin{array}{ccc} \; & 1 & \;\mathrm{if}\;p_{i}\in {\mathrm{ARH}}_{j}\\ \; & 0 & \;\mathrm{otherwise} \end{array}\right.$$

**Figure 4:**
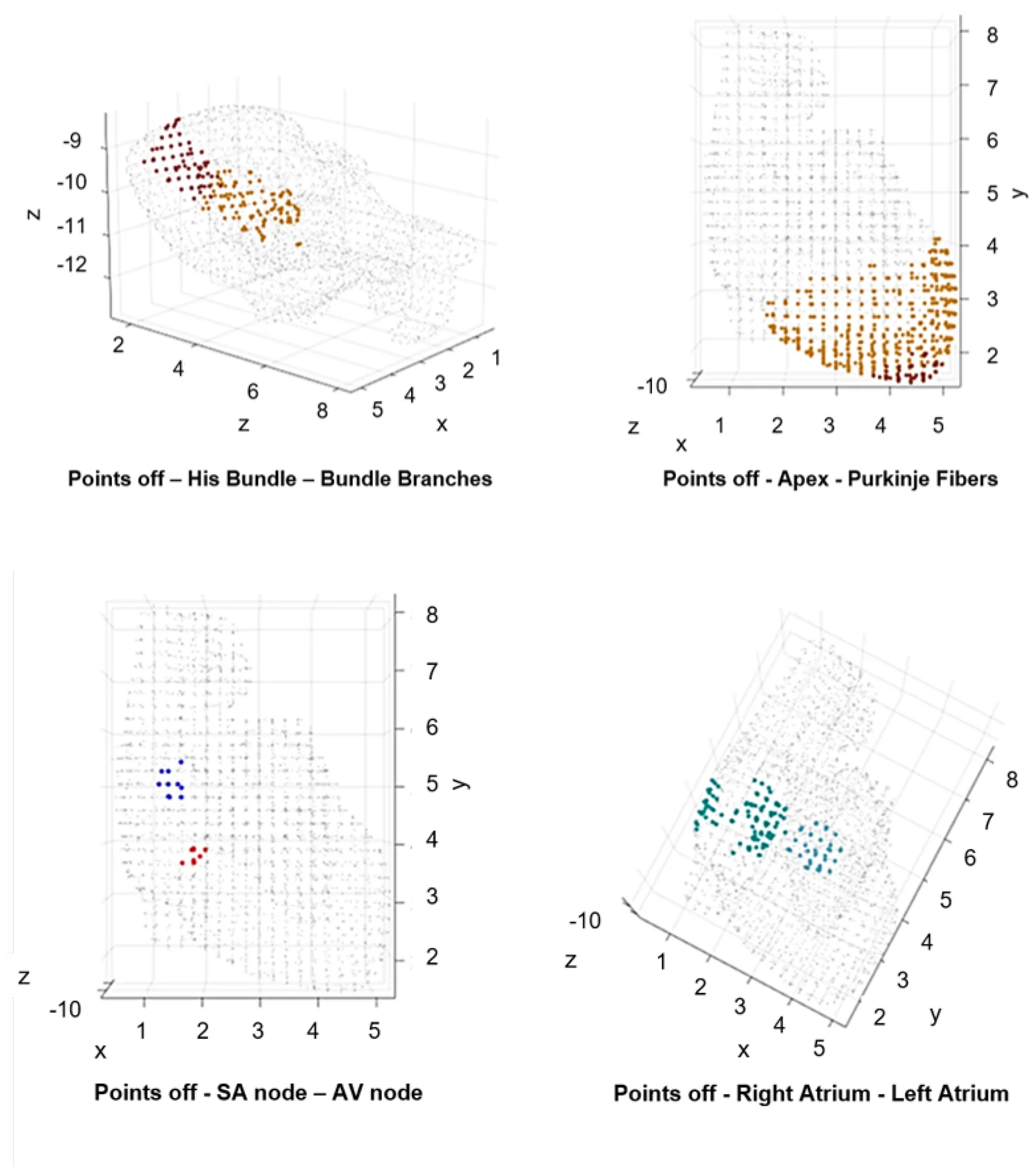
Anatomical segmentation of the heart point cloud into the eight regions used to define the activation space matrix.

For this proof-of-concept implementation, one operator manually partitions the heart point cloud into $N_{\mathrm{ARH}}=8$ predefined anatomical regions, as shown in Figure 4: the sinoatrial (SA) node, atrioventricular (AV) node, right atrium, left atrium, His bundle, bundle branches, apex, and Purkinje-fiber region. The segmentation is performed once on a generic heart geometry and is reused for all ECG recordings. The region assignments are therefore not derived from patient-specific electrophysiological measurements but provide a model-driven spatial support for mapping ECG-derived timing and amplitude information.

### 3.4. Vertex-Time Signal Analysis

**Figure 5:**
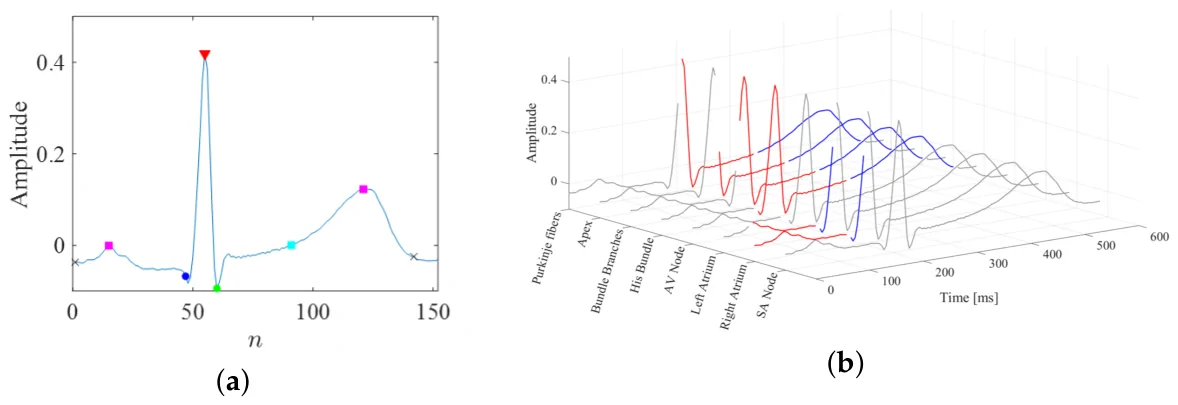
(**a**) Eigenbeat The markers identify the P-wave onset, P peak, Q point/QRS onset, R peak, S point, T-wave onset, T peak, and T-wave offset. (**b**) Deterministic association of the landmark-defined intervals with the predefined anatomical regions. Red and blue lines indicate depolarization and repolarization intervals, respectively. Gray lines indicate inactive intervals. The illustrated sequence shows the adopted model-driven mapping.

This stage associates the eigenbeat ${\hat{s}}_{\mathrm{beat}}$ with predefined anatomical regions to construct two vertex-time signals on the cardiac graph. The Ideal Vertex Time (IVT) signal encodes trigger, depolarization, and repolarization intervals, whereas the Eigen Vertex Time (EVT) signal maps eigenbeat amplitudes onto the active anatomical regions. The P wave mainly reflects atrial depolarization, the QRS complex reflects ventricular depolarization, and the T wave reflects ventricular repolarization. These surface-ECG events provide temporal information but do not uniquely determine the three-dimensional origin of cardiac activation. The eigenbeat ${\hat{s}}_{\mathrm{beat}}$ is characterized by eight temporal landmarks used to delimit the P-wave, QRS-complex, and T-wave phases, as shown in Figure 5a. Using this temporal information, we introduce the activation time matrix representing the temporal activation of each anatomical region.

We define the activation time matrix $B_{\mathrm{time}}$ as a binary matrix with $N_{\mathrm{ARH}}$ rows and $N_{s}$ columns, where $N_{s}$ denotes the number of time samples of the binary activation signal:

(4) $$B_{\mathrm{time}}={\left[b_{1}^{\left(\mathrm{time}\right)},\ldots ,b_{N_{\mathrm{ARH}}}^{\left(\mathrm{time}\right)}\right]}^{T}$$

(5) $$b_{j}^{\left(\mathrm{time}\right)}={\left[b_{j}^{\left(\mathrm{time}\right)}\left(t_{1}\right),\ldots ,b_{j}^{\left(\mathrm{time}\right)}\left(t_{N_{s}}\right)\right]}^{T}$$

(6) $$b_{j}^{\left(\mathrm{time}\right)}\left(t_{i}\right)=\left\{\begin{array}{ccc} \; & 1 & \;\mathrm{if}\;t_{i}\in {\mathrm{ARH}}_{j}\\ \; & 0 & \;\mathrm{otherwise} \end{array}\right.$$

Figure 5 shows the highlighted samples of the eigenbeat ${\hat{s}}_{\mathrm{beat}}$ for each cardiac region, where the sinoatrial and atrioventricular nodes act as the physiological triggers of atrial and ventricular depolarization, respectively. Red lines indicate depolarization events, while blue lines represent repolarization events. Within the adopted model, atrial depolarization is associated with the P wave, while atrial repolarization begins at the onset of the QRS complex. Ventricular depolarization is associated with the QRS complex, whereas ventricular repolarization is represented by the T wave.

We introduce the global activation matrix B:

(7) $$B=B_{\mathrm{space}}\cdot B_{\mathrm{time}},$$

which is a binary matrix $N_{\mathrm{PC}}$ by $N_{s}$ combining the spatial and temporal activation patterns of the two activation matrices $B_{\mathrm{space}}$ and $B_{\mathrm{time}}$. The ECG-to-geometry mapping is therefore performed through the following deterministic sequence:

1. The ECG beats are detected, segmented, and aligned at the R peak;
2. The eigenbeat and its P-QRS-T landmarks are estimated;
3. Each landmark-defined temporal interval is associated with one or more predefined anatomical regions through $B_{\mathrm{time}}$;
4. Each point-cloud vertex is assigned to one anatomical region through $B_{\mathrm{space}}$;
5. The product $B=B_{\mathrm{space}}B_{\mathrm{time}}$ transfers the region-level temporal model to all vertices belonging to each region;
6. IVT labels or EVT eigenbeat amplitudes are applied to the active vertices at each time sample.

Two graph signals are then defined to encode the depolarization/repolarization events and the eigenbeat amplitudes, respectively. The first is the labeling time signal $d^{\left(\mathrm{IVT}\right)}$, whose *i*-th coefficient is given by

(8) $$d_{i}^{\left(\mathrm{IVT}\right)}=\left\{\begin{array}{ccc} \; & 3 & i\in \mathrm{Repolarization}\;\mathrm{Interval}\\ \; & 2 & i\in \mathrm{Depolarization}\;\mathrm{Interval}\\ \; & 1 & i\in \mathrm{Trigger}\;\mathrm{Interval}\\ \; & 0 & \;\mathrm{otherwise} \end{array}\right.$$

and the amplitude time signal

(9) $$d^{\left(\mathrm{EVT}\right)}={\hat{s}}_{\mathrm{beat}}.$$

The corresponding vertex-time signals are represented by the matrices

(10) $$S_{\mathrm{IVT}}=B\cdot \mathrm{diag}\left(d^{\left(\mathrm{IVT}\right)}\right),$$

(11) $$S_{\mathrm{EVT}}=B\cdot \mathrm{diag}\left(d^{\left(\mathrm{EVT}\right)}\right),$$

The vertex-time IVT signal $S_{\mathrm{IVT}}$ encodes the temporal structure of depolarization and repolarization events, while the EVT signal $S_{\mathrm{EVT}}$ captures the corresponding amplitude information derived from the eigenbeat. The two signals describe different aspects of the same representative beat and can therefore be considered jointly, as discussed in Section 4.2.

### 3.5. Graph-Spectral Analysis

For both the IVT vertex-time signal $S_{\mathrm{IVT}}$ and the EVT vertex-time signal $S_{\mathrm{EVT}}$, the Graph Fourier Transform is computed column-wise.

To this aim, given the heart point cloud, both vertices and faces are extracted to construct a triangulated surface representation of the cardiac geometry. The vertices are defined as the coordinates of each point $V=\left\{p_{i}={\left[p_{ix},p_{iy},p_{iz}\right]}^{T};i=1,\ldots ,N_{\mathrm{PC}}\right\}$, while the faces are obtained by reconstructing a triangulated surface from the point cloud, resulting in the set of triangular faces through a surface reconstruction procedure [25]. Specifically, given the set of vertices V, a triangulated surface is constructed, and the set of triangular faces is defined as $T=\left\{t_{i}=\left(p^{\left(1\right)},p^{\left(2\right)},p^{\left(3\right)}\right);\;i=1,\ldots ,N_{T}\right\}$, where each triangular face is formed by three vertices in V. The resulting heart mesh is denoted as M=(V,T).

To enable spectral processing, the mesh is modeled as a weighted graph, where each vertex corresponds to a node and edges are induced by the mesh connectivity. The weight (adjacency) matrix $W\in R^{N\times N}$ encodes the similarity between connected vertices, with entries $w_{ij}$ defined based on geometric proximity or feature similarity. The corresponding degree matrix is $D=\mathrm{diag}(d_{1},\ldots ,d_{N})$, where $d_{i}=\sum_{j}w_{ij}$.

Based on this graph representation, the Laplacian matrix L is defined as the symmetric normalized graph Laplacian: $L=I-D^{-\frac{1}{2}}WD^{-\frac{1}{2}},$ where I denotes the identity matrix. This formulation corresponds to the normalized graph Laplacian commonly used in spectral graph theory.

From the eigen-analysis

(12) $$L=U\Lambda U^{T},$$

the Graph Fourier Transform basis U is computed. We denote the Graph Fourier Transform of $S_{\mathrm{IVT}}$ and $S_{\mathrm{EVT}}$ as

(13) $$\begin{array}{cc} F^{\left(\mathrm{IVT}\right)} & \;=\left[f_{0}^{\left(\mathrm{IVT}\right)}\cdots f_{N_{s}-1}^{\left(\mathrm{IVT}\right)}\right],\\ F^{\left(\mathrm{EVT}\right)} & \;=\left[f_{0}^{\left(\mathrm{EVT}\right)}\cdots f_{N_{s}-1}^{\left(\mathrm{EVT}\right)}\right], \end{array}$$

being $f_{i}^{\left(\mathrm{EVT}\right)}=Us_{i}^{\left(\mathrm{EVT}\right)}$, $f_{i}^{\left(\mathrm{IVT}\right)}=Us_{i}^{\left(\mathrm{IVT}\right)}$, for $i=1,\ldots ,N_{s}$.

For graph-spectral feature extraction, only the first $N_{\mathrm{low}}$ low-frequency coefficients and the last $N_{\mathrm{high}}$ high-frequency coefficients are retained, namely those corresponding to the first $N_{\mathrm{low}}$ rows and the last $N_{\mathrm{high}}$ rows of $F^{\left(\mathrm{IVT}\right)}$ and $F^{\left(\mathrm{EVT}\right)}$.

The retained graph-spectral coefficients form the feature vectors used in the classification experiments described in Section 4. The spectral analysis relies on the fact that ECG-derived activity is mapped onto anatomically contiguous cardiac regions so that the vertex-time signals preserve a degree of spatial continuity, without abrupt, unstructured spatio-temporal changes over the cardiac mesh. Locally adaptive or region-aware graph operators [26] may also be adopted to better characterize localized discontinuities. The corresponding vertex-time signals are also provided in the IoT-XR pipeline introduced in Section 5.

## 4. Classification Experiments

The experimental evaluation comprises two distinct analyses. Section 4.2 examines the effect of graph-spectral coefficient selection on IVT, EVT, and combined IVT + EVT features for valence classification. Section 4.3 evaluates IVT and EVT features for valence and arousal classification under class imbalance and includes an additional analysis of the effect of heart-mesh resolution. Because the two analyses use different feature settings, classifiers, validation protocols, and evaluation metrics, their results are presented separately.

### 4.1. Dataset and Experimental Setup

The YAAD dataset serves as a proof-of-concept benchmark for assessing whether the vertex-time features retain information relevant to affective-state classification. The original set considered in this work contains 406 ECG recordings from 25 participants. It comprises 252 recordings from 12 participants belonging to the multimodal subset and 154 recordings from 13 different participants belonging to the single-modal subset. Twelve recordings are excluded because of ECG segmentation or landmark detection failures, resulting in 394 valid recordings used in the experimental evaluation. The retained recordings are associated with binary arousal and valence labels. The class distributions are 130 high-arousal versus 264 low-arousal recordings and 135 high-valence versus 259 low-valence recordings. The ECG signals in the YAAD dataset were acquired at a sampling rate of 256 Hz. According to the dataset authors [27], the ECG recordings were pre-filtered before storage using a second-order Chebyshev low-pass filter with a cutoff frequency slightly below the Nyquist frequency and a second-order Chebyshev high-pass filter with a cutoff frequency of 0.5 Hz in order to reduce environmental noise and muscle-related interference. During acquisition, external interference and motion artifacts were further limited by removing mobile phones and other electronic devices from the recording environment and instructing participants to remain still and avoid unnecessary movements. For the experiments presented in this work, each ECG recording is truncated to the first five seconds and baseline-centered before feature extraction. Table 2 summarizes the main characteristics of the YAAD ECG data used in the experimental evaluation.

All cross-validation folds are defined at the recording level and stratified by class labels. Recordings are not grouped by participant; therefore, recordings from the same participant may occur in both the training and test sets. This aligns with the current literature [11,27], and it reflects what is done in the application, where data-driven e-health monitoring algorithms are tuned to the patient under concern. The main objectives, classifiers, validation strategies, and evaluation metrics adopted in the two experimental analyses are summarized in Table 3.

### 4.2. Spectral Configuration Analysis

The first experimental analysis investigates the influence of graph-spectral coefficient selection on the discriminative capability of the vertex-time features. A binary high-valence versus low-valence classification task is performed on the YAAD dataset. The features are extracted according to the procedure described in Section 3.5.

Three feature configurations are considered: IVT, EVT, and their concatenation, denoted as IVT+EVT. IVT encodes the temporal ordering of trigger, depolarization, and repolarization events, whereas EVT associates the eigenbeat amplitude profile with the predefined cardiac regions. For both representations, the Graph Fourier Transform is computed on the cardiac mesh.

Feature vectors are constructed by selecting combinations of $N_{\mathrm{low}}$ low-frequency and $N_{\mathrm{high}}$ high-frequency Laplacian components. These combinations are explored through a grid search to assess the contribution of both global and local graph-spectral patterns.

Features extracted from the two ECG channels are concatenated and classified using a linear support vector machine. Performance is evaluated through stratified five-fold cross-validation. The five-fold protocol follows the recording-level splitting strategy described in Section 4.1. Accuracy, balanced accuracy, and macro F1-score are reported.

The results show limited variation across the tested spectral configurations. IVT provides the most balanced performance, with the best configuration ($N_{\mathrm{low}}=30$, $N_{\mathrm{high}}=20$) achieving an accuracy of approximately 0.61, a balanced accuracy of 0.539, and a macro F1-score of 0.538. EVT reaches a higher raw accuracy, up to 0.658, but a lower balanced accuracy, indicating greater sensitivity to class imbalance. The combined IVT + EVT representation achieves a maximum balanced accuracy of 0.524 for ($N_{\mathrm{low}}=10$, $N_{\mathrm{high}}=10$). Under the tested configurations, feature concatenation does not improve the best balanced accuracy obtained with IVT alone.The combined configuration therefore does not improve balanced accuracy over IVT alone (0.524 versus 0.539). In the reported analysis, concatenating IVT and EVT features does not provide evidence of complementary discriminative information, and the two representations are better regarded as alternatives whose relative effectiveness depends on the target task.

### 4.3. Imbalanced Valence and Arousal Classification

The second analysis evaluates IVT and EVT features on two binary classification tasks: high versus low valence and high versus low arousal. For each ECG recording, the first five seconds are retained and the baseline mean is removed. Features are extracted using the *heart2k* point cloud and a fixed spectral configuration with $N_{\mathrm{low}}=10$ and $N_{\mathrm{high}}=10$.

Classification is performed using stratified ten-fold cross-validation. The ten-fold evaluation follows the recording-level, class-stratified splitting strategy described in Section 4.1. A cubic support vector machine is used for valence classification, whereas a subspace KNN classifier is used for arousal classification.

Because of class imbalance, accuracy, weighted precision, recall, and F1-score are reported. Precision, recall, and F1-score are computed by weighting the class-specific values according to the corresponding class support.

As reported in Table 4, IVT achieves the highest performance for valence classification, whereas EVT provides the strongest results for arousal classification. The comparison indicates that the relative performance of IVT and EVT depends on the target task.

#### Effect of Mesh Resolution

Within the imbalanced valence and arousal classification analysis, the effect of heart-mesh resolution is evaluated using geometries with N=2000, N=18,000, and N=92,000 vertices. IVT is evaluated on the 2000 and 18,000 vertex geometries, whereas EVT is evaluated on the 18,000 and 92,000 vertex meshes. IVT achieves an accuracy of 67.04% on both the 2000 and 18,000 vertex geometries. EVT achieves 68.27% on the 18,000 vertex mesh and 67.42% on the 92,000 vertex mesh. Among the tested configurations, EVT on the 18,000 vertex mesh provides the highest raw accuracy. In applications to individual monitoring, either IVT or EVT or a combination of both can be selected depending on the performance observed for the specific subject.

### 4.4. Summary of Classification Results

In the spectral-configuration analysis, IVT achieves the highest balanced accuracy, whereas EVT reaches the highest raw accuracy. In the imbalanced valence and arousal analysis, IVT performs best for valence classification, while EVT performs best for arousal classification. Within the same analysis, EVT on the 18,000-vertex mesh achieves the highest raw accuracy among the tested geometric configurations. The relative performance of IVT and EVT therefore depends on the classification task, evaluation metric, and mesh configuration.

The limited variation observed across the tested spectral settings suggests moderate sensitivity to coefficient selection within the evaluated range. The mesh-resolution results show similarly small accuracy differences across the available configurations; however, the incomplete pairing of IVT and EVT across identical meshes prevents a controlled assessment of geometric sensitivity.

Overall, IVT and EVT encode different aspects of the representative ECG beat, while further analysis is needed to establish if they yield complementary discriminative information. The classification experiments assess the discriminative capability of the proposed vertex-time representation rather than optimize a task-specific classifier or provide an exhaustive comparison with conventional ECG features. No comparison against conventional ECG descriptors, such as time-domain morphological features, heart-rate variability indices, or standard interval measurements, was carried out. The reported results therefore indicate that the vertex-time representation retains discriminative information, while its added value relative to conventional ECG features remains to be investigated. For valence classification, IVT achieves 76.92% accuracy and 77.35% weighted precision without dedicated hyperparameter optimization. Additional experiments using different fractions of the training data showed consistent feature behavior, although they are not reported here for conciseness. For subject-specific monitoring applications, future work should include direct comparison with conventional ECG descriptors and learning-curve analysis.

## 5. IoT-XR Prototype

**Figure 6:**
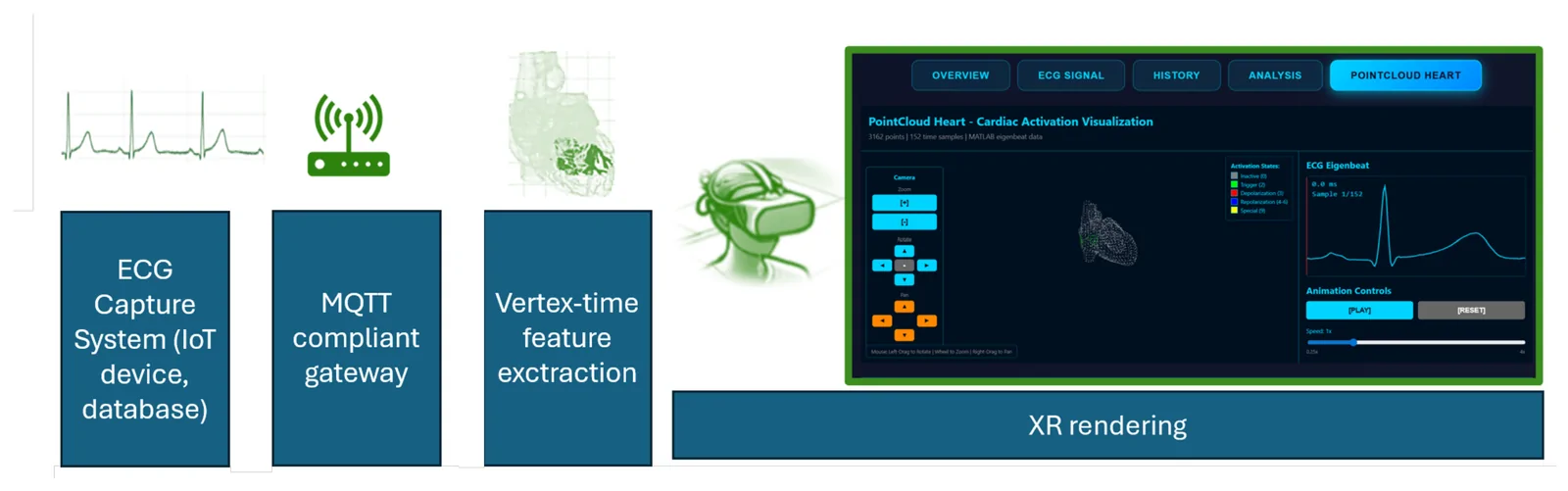
End-to-end architecture of the proof-of-concept IoT-XR prototype, from ECG streaming and cloud-based vertex-time processing to web-based immersive visualization.

Figure 6 illustrates the end-to-end architecture of the implemented IoT–XR prototype, from ECG streaming to immersive visualization.

### 5.1. End-to-End Architecture

The implemented prototype connects ECG data sources, MQTT-based communication, Node-RED middleware, cloud processing, and a web-based XR interface. As shown in Figure 6, the vertex-time representation acts as an intermediate data model between physiological signal processing and immersive visualization.

### 5.2. IoT Data Pipeline

The architecture supports ECG acquisition from wearable devices or simulated smartwatch sources. The resulting ECG streams are transmitted through the MQTT publish-subscribe protocol. In the current implementation, publicly available ECG recordings are replayed as continuous streams to emulate wearable data acquisition. The original YAAD recordings are sampled at 256 Hz, whereas the prototype emits 128 samples per second. Therefore, a nominal 5 s processing window in the prototype contains 640 samples, corresponding to 2.5 s of the original ECG recording. Consequently, the current prototype playback does not preserve the original physiological time scale and should be regarded as a demonstrative streaming configuration rather than as a physiologically accurate real-time reproduction. This prototype configuration is distinct from the classification experiments, where each 5 s segment contains 1280 samples at the original 256 Hz sampling rate and therefore corresponds to 5 s of physiological ECG data.

Incoming ECG packets are handled through a Node-RED middleware layer providing buffering, protocol abstraction, RESTful APIs, and modular communication between producers and consumers. Cloud processing performs online R-peak detection, R-R interval analysis, and graph-based vertex-time feature extraction described in Section 3.5.

### 5.3. XR Visualization

The extracted vertex-time features are streamed to a web-based XR interface that supports continuous visualization of ECG traces, derived heart-rate information, and spatially distributed cardiac activity. The interface links the temporal ECG waveform to the corresponding regions of the heart point cloud, allowing users to inspect both signal dynamics and their model-driven spatial representation within the same immersive environment.

### 5.4. Latency and Bandwidth Characterization

The IoT-XR processing chain was characterized under co-located experimental conditions by separately measuring the main communication and processing stages. Table 5 reports the resulting stage-wise delays and the associated communication data rates. These measurements characterize the software and protocol chain and do not include the additional latency of a specific wireless or radio access network. The measured communication and processing stages indicate that an ECG packet can travel from the publishing source to the visualization client in approximately 15 ms under the tested co-located conditions, with the vertex-time/GFT computation adding less than 1 ms per analysis window. However, transport and processing latency do not dominate the freshness of the displayed information in the current configuration.

The source publishes one packet per second and the visualization client refreshes at 1 Hz. Therefore, the software interface is updated at a nominal 1 s cadence. This cadence refers to prototype playback time and does not preserve the physiological time scale of the original 256 Hz ECG recordings. Transport and processing latency are substantially shorter than this nominal refresh interval under the tested co-located conditions. This delay is primarily determined by the selected acquisition and refresh cadence rather than by communication or graph processing. In terms of bandwidth, in the current prototype configuration, the ECG uplink transfers approximately 1.26 kB per message at one message per second, corresponding to approximately 10 kbit/s per monitored device. The cloud-to-visualization link transfers approximately 8.1 kB per refresh at 1 Hz, corresponding to approximately 65 kbit/s per client. The 6.2 MB heart point cloud and activation sequence are transferred only once at session initialization and remain resident at the client. Therefore, the three-dimensional geometry does not introduce a recurring network load during monitoring. These measurements allow us to characterize the processing and protocol chain under co-located conditions and may differ by deployment over a specific wireless network. Controlled experiments under packet loss, and bandwidth constraints are outside the scope of this paper, and are left for future work. In the current QoS-0 configuration, each MQTT message is self-contained and carries approximately one second of ECG data. Therefore, loss of an individual packet affects the corresponding ECG segment but does not corrupt the state of subsequent packets. However, packet loss and degraded network conditions in a real environment may affect the overall system performance, as discussed in [28].

## 6. Discussion and Limitations

The results show that a model-driven mapping of ECG-derived information onto a three-dimensional cardiac graph can support both graph-spectral analysis and immersive visualization. IVT and EVT provide distinct descriptions of cardiac timing and amplitude, while the IoT-XR prototype demonstrates how the same representation can be integrated into an end-to-end monitoring architecture.

The spatial mapping is based on a heart geometry manually partitioned into eight defined regions, providing a model-driven support for the vertex-time representation rather than a patient-specific reconstruction. A natural extension is the use of patient-specific cardiac geometries reconstructed from medical imaging. Recent work has demonstrated the feasibility of whole-heart 3D reconstruction from sparse echocardiographic views using CT-derived supervision, supporting the possibility of incorporating personalized cardiac geometries in future implementations [29].

The classification experiments use the relatively small and imbalanced YAAD affective-state dataset, which was not designed for cardiac diagnosis. Moreover, cross-validation is performed at the recording level, allowing recordings from the same participant to appear in both training and test folds. The reported performance should therefore be interpreted as proof-of-concept feature discriminability rather than evidence of clinical validity or generalization to unseen participants. Since no conventional ECG feature baseline was evaluated, no claim of superiority over established ECG descriptors is made or supported by these experiments. In fact, the present study demonstrates the discriminative potential of the proposed vertex-time features. They can therefore provide additional representations of ECG information that may be integrated into a broader feature set and considered for selection in a patient-specific training framework. In the reported experiments, the joint use of IVT and EVT features did not improve balanced accuracy over IVT alone. Therefore, while IVT and EVT provide distinct representations of temporal and amplitude-related ECG information, their potential complementary contribution to classification remains to be investigated in broader feature sets and patient-specific training frameworks. Although this protocol does not assess subject-independent generalization, recording-level evaluation may also be relevant to personalized monitoring scenarios in which previous recordings from the same patient are available for model adaptation. Participant-independent validation remains necessary to assess generalization to unseen subjects.

Extension to pathological ECGs, including arrhythmic conditions, would require pathology-specific adaptation of the current P-QRS-T-based preprocessing, since characteristic landmarks may be altered or absent. Accordingly, beat segmentation, landmark detection, and temporal activation intervals should be retuned according to the morphology of the target pathology. In practical scenarios, the low- and high-frequency graph components to be retained should be tuned to the subject under concern [30], since their optimal number and combination depend on the target task and the pathology. Furthermore, future work can address joint spatio-temporal segmentation and persistent component extraction, so as to extend results related to temporal signals [31].

The IoT-XR implementation currently relies on replayed public ECG recordings rather than direct acquisition from a physical wearable device. The current prototype uses a nominal playback rate that does not systematically preserve the original sampling rate of the YAAD recordings, and it is adopted here to assess the feasibility of the software integration of vertex-time generation, communication, and XR rendering. The stage-wise latency measurements characterize the processing and protocol chain under co-located conditions, but they may differ over different real wireless access networks. The reported XR client delays refer to data preparation and per-step vertex updates in a web browser; they could further vary depending on the actual rasterization adopted over a specific head-mounted display. Beyond monitoring, the proposed immersive vertex-time representation could be investigated as an educational tool for healthcare personnel, supporting interactive visualization of cardiac anatomy and ECG-related activation patterns in XR environments [8,32]. To sum up, this work introduces a novel ECG vertex-time representation and opens several directions for future development either from the signal processing point of view: (i) improving the resilience to artifacts due to physical wearable sensors, and (ii) investigating the impact of subject-specific heart geometries and ECG pattern features, or networking aspects, i.e., feasibility in a specific wireless environment or for headset-specific performance evaluation.

## 7. Conclusions

This study introduced a vertex-time framework that maps ECG-derived timing and amplitude information onto a suitably segmented three-dimensional heart point cloud. The vertex-time representation is model-driven, i.e., it is segmented in fixed regions associated to predefined temporal activation sequences. Therefore, it provides a structured analysis and visualization support rather than a reconstruction of cardiac activation built through electroanatomical measurement. The proof-of-concept results on the YAAD dataset show that the proposed features retain discriminative information for valence and arousal classification. They do not establish an advantage over conventional ECG features, which were not evaluated in this work; rather, they provide additional representations of ECG information that may be integrated in future patient-specific classification frameworks. The IVT and EVT formulations provide distinct graph signals encoding temporal and amplitude-related information, respectively, and support graph-spectral feature extraction and affective-state classification. The same representation was integrated into an end-to-end IoT-XR prototype based on MQTT communication, Node-RED middleware, cloud processing, and a web-based immersive interface. This integration shows how ECG-derived graph signals can serve not only as classification features but also as spatially organized inputs for low-latency immersive visualization of cardiac activity. This feasibility was assessed on replayed recordings with co-located services and not on live wearable acquisition, preserving the physiological time scale.

The proposed vertex-time framework can be further investigated across different ECG monitoring scenarios. Future work is therefore needed in different directions, such as data-driven inference of the vertex-time activation signal, subject-specific parameter tuning, outlier-resilient data processing for physical wearable devices, as well as real-time, headset-specific performance evaluation of the IoT-XR pipeline under different network conditions.

## Figures and Tables

**Table 1:** Comparison of representative approaches related to ECG analysis, non-Euclidean cardiac representations, and XR-based visualization.

Work	Main Focus	Strengths	Novelty of This Work
Di Salvo et al. [12]	Graph-based ECG analysis	Multiscale non-Euclidean ECG features for physiological-state classification	Mapping of the signal into 3D heart geometry and integration into IoT-XR visualization pipeline.
Kong and Shadden [13]	3D heart mesh generation	Realistic cardiac geometries derived from patient imaging	Integration of the ECG temporal component into a vertex-time signals.
Yang and Gao [14]	Point-cloud cardiac analysis	Demonstrates the effectiveness of point-cloud representations for cardiac classification and segmentation	Introduction of the ECG-derived vertex-time signals in XR monitoring.
Amara et al. [19]	ECG analysis and XR visualization	Combines arrhythmia-related information with immersive cardiac visualization	Integration of visualization with ECG vertex-time feature extraction.
Marteau et al. [20]	Immersive clinical-data visualization	Supports spatial visualization and interaction with multimodal clinical data	Introduction of the ECG-derived vertex-time features for ECG monitoring.
Fejzic et al. [23]	IoT-XR ECG streaming prototype	Characterizes end-to-end IoT latency and MQTT-based streaming for ECG-to-XR visualization	Introduction of the novel ECG-derived vertex-time signals in XR monitoring.
Silvi et al. [11]	Wavelet-graph ECG representation	Subband wavelet-graph encoding for affective and health-state classification	Introduction of spatial information in the ECG temporal analysis.

**Table 2:** Summary of the YAAD ECG data used in this study (further details available in [27]).

Characteristic	Value
Original recordings	406
Valid recordings used	394
Participants	25
Sampling rate	256 Hz
Analysis duration	First 5 s of each recording
Arousal classes	130 high/264 low
Valence classes	135 high/259 low
Dataset filtering	2nd-order Chebyshev LPF and HPF
HPF cutoff	0.5 Hz
Additional preprocessing	Baseline centering

**Table 3:** Summary of the classification analyses.

Analysis	Objective	Classifier/Validation	Metrics
Spectral configuration	Evaluate IVT, EVT, and IVT + EVT spectral features for valence classification.	Linear support vector machine (SVM); stratified five-fold cross-validation (CV)	Accuracy; balanced accuracy; macro F1
Valence and arousal	Compare IVT and EVT under class imbalance and assess the effect of mesh resolution.	Cubic SVM for valence; subspace k-nearest neighbors (KNN) for arousal; stratified ten-fold CV	Accuracy; weighted precision, recall, and F1

**Table 4:** Weighted classification performance of IVT and EVT graph-spectral features for binary valence and arousal classification (%).

Task	Accuracy	Precision	Recall	F1-Score
Valence (EVT)	74.36	73.79	74.36	73.87
Arousal (EVT)	71.80	71.30	71.79	71.50
Valence (IVT)	76.92	77.35	76.92	77.09
Arousal (IVT)	66.67	63.13	66.67	62.50

**Table 5:** Stage-wise characterization of the IoT-XR processing chain under co-located experimental conditions. Communication and XR-client values were measured on the running prototype, while the vertex-time projection latency was separately benchmarked Python in Python 3.11.9 on the same 3162-point cardiac geometry. The Graph Fourier basis is point-cloud-dependent and can be precomputed.

Stage	Measured Latency/Timing	Notes
MQTT publish-to-subscriber delivery	0.6 ms median; 2.3 ms mean; 21 ms p95	QoS 0; 1.25 kB payload; 50 messages
Middleware buffering and REST exposure	1.5 ms median	8.1 kB REST response
Cloud aggregation and REST gateway	12.6 ms median; 17.0 ms p95	Includes middleware hop
Front-end proxy delivery to client	13.1 ms median	Includes the preceding communication hops
Vertex-time/GFT projection	0.70 ms per 640-sample prototype window	3162 vertices; 40 retained coefficients; 6080 features
Graph Fourier basis computation	0.16 s, precomputed	Mesh-Laplacian eigendecomposition
Activation-space/time computation	4 ms	3162 vertices
Heart geometry transfer	141 ms, once per session	6.2 MB transferred to the client
UTF-8 decoding and parsing	142 ms, once per session	11 ms decoding + 131 ms parsing
Vertex-buffer construction	0.5 ms, once per session	3162 vertices
XR vertex-color update	0.2 ms median; 5.4 ms p95	3162 vertices; nominally 20 activation steps/s
Acquisition and visualization refresh cadence	1 s	Dominant contribution to displayed-data freshness

## Data Availability

The data used in this study are available in the public domain: “Young Adult’s Affective Data (YAAD) Using ECG and GSR Signals”, https://data.mendeley.com/datasets/g2p7vwxyn2/4 accessed on 24 September 2026.
